# High-dose IV acetaminophen reduces delirium risk in older adults with acute abdominal conditions: a retrospective cohort study

**DOI:** 10.1186/s40780-025-00462-1

**Published:** 2025-07-01

**Authors:** Masayuki Saito, Nanaha Nishiwaki, Yoshihito Nakashima, Eisei Hori, Tadashi Suzuki, Tomoya Tachi, Toshihiko Ichihara

**Affiliations:** 1 Department of Pharmacy, Tosei General Hospital, 160 Nishi-Oiwake-Cho, Seto, Aichi 489-8642 Japan; 2 Department of Emergency Medicine, Tosei General Hospital, 160 Nishi-Oiwake-Cho, Seto, Aichi 489-8642 Japan; 3 Department of Clinical Pharmacy, Graduate School of Pharmaceutical Sciences, Nagoya City University, 3-1 Tanabe-Dori, Mizuho-Ku, Nagoya, 467-8603 Japan

**Keywords:** Acetaminophen, Delirium, Acute abdomen, Emergency Department, Elderly patients

## Abstract

**Background:**

Delirium, a significant complication in older patients, often occurs during hospitalization and is associated with poor clinical outcomes. Effective strategies to prevent delirium are essential, particularly in emergency department (ED) settings where older patients frequently present with acute abdominal conditions. This study evaluated the impact of high-dose intravenous acetaminophen (≥ 1,000 mg) on the onset of delirium in older patients.

**Methods:**

This retrospective cohort study included 411 patients aged 70 years or older diagnosed with acute abdomen at the ED of Tosei General Hospital from October 2015 to December 2022. Patients were divided into high-dose (≥ 1,000 mg/administration) and low-dose (< 1,000 mg/administration) groups based on acetaminophen dosage. Multivariate logistic regression analysis was performed to adjust for confounding factors, including neurodegenerative diseases, sensory impairments, and serum albumin levels.

**Results:**

Of the 411 patients included in this study, 53 (12.9%) developed delirium during hospitalization, with the high-dose acetaminophen group demonstrating a significantly lower risk of delirium onset than that of the low-dose group (odds ratio: 0.391; 95% confidence interval: 0.193–0.791). Multivariate logistic regression analysis confirmed the protective effect of high-dose acetaminophen treatment after adjusting for potential confounding factors, suggesting this treatment protocol as a promising therapeutic approach for preventing delirium in older patients with acute abdominal conditions.

**Conclusions:**

High-dose intravenous acetaminophen may effectively reduce the risk of delirium onset in older patients hospitalized with acute abdomen. These findings suggest a valuable role for high-dose acetaminophen in improving patient outcomes and reducing the burden of delirium in emergency and hospital care.

**Trial registration:**

Retrospectively registered.

## Background

Delirium is a mental and behavioral disorder caused by physical illness, with older patients, particularly at high risk of developing it during hospitalization. In Japan, where the aging population is rapidly increasing, emergency departments (ED) often act as the primary point of admission for older patients [1, 2]. Studies indicate that ED visits by elderly individuals increase the risk of developing delirium (Annual Report on the Ageing Society 2023 [2]), with reports indicating that 8–10% of those admitted through the ED develop delirium during their hospital stay [3]. This makes delirium a critical concern in emergency care. The onset of delirium complicates the treatment of underlying diseases and increases the burden on healthcare providers and families, thereby leading to higher mortality rates and elevated risks of infection [4]. Therefore, preventing delirium onset is crucial for improving patient outcomes.

Various factors contribute to the development of delirium, with pain being more significant [5]. Pain is a significant source of distress for patients and a risk factor for delirium onset, necessitating appropriate pain management [6]. Acetaminophen is an extensively used analgesic globally, particularly in the ED, where it is frequently administered to patients with acute abdomen requiring rapid pain relief [7]. Current guidelines recommend an intravenous dose of 1,000 mg of acetaminophen for patients with acute abdomen, which is generally considered high. Typically, acetaminophen dosage is adjusted based on body weight, with the package insert specifying a maximum dose of 15 mg/kg for adults weighing less than 50 kg [8]. Hence, a 1,000-mg dose for an adult weighing 50 kg is categorized as high.

While acetaminophen is effective for pain management, it reportedly affects mental status [9]. The proposed mechanism involves the passage of acetaminophen through the blood–brain barrier, allowing it to act on neural cells in the brain [10]. However, the impact of high-dose acetaminophen on the onset of delirium remains unclear.

This study evaluated whether the administration of high-dose acetaminophen in older patients diagnosed with acute abdomen in the ED can suppress the onset of delirium during hospitalization.

## Methods

### Study design and participants

This retrospective cohort study involved patients aged 70 years or older diagnosed with acute abdominal conditions in the ED of Tosei General Hospital who received intravenous acetaminophen between October 2015 and December 2022.

### Data collection

Data were retrospectively collected from electronic medical records. In this study, delirium was defined as cases in which medical staff recorded a diagnosis of delirium in the electronic medical records. Experienced medical staff assessed delirium based on observed changes in mental status, attention deficits, hallucinations, or illusions. The evaluation period for delirium was set within 7 days of hospital admission. Since delirium commonly develops within the first few days of hospitalization and the average length of stay at Tosei General Hospital is approximately 10 days, we established this evaluation period while considering the potential influence of other factors on delirium onset. The items selected for analysis were based on risk factors for delirium reported in previous studies, focusing on information that can be promptly obtained in the ED to facilitate clinical decision-making. In the ED, patient information is often uncertain, requiring rapid decision-making with limited data. Consequently, data derived from initial assessments in the ED are considered more critical than detailed information obtained after admission. This study investigated the following items that can be assessed early in the ED: age, sex, living situation [11], history of neurodegenerative diseases/dementia sensory impairments (vision/hearing) [12], history of delirium [11, 12], use of drugs associated with the risk of delirium [11, 12], use of six or more medications [11], serum bilirubin [11, 12], albumin [11, 12], blood urea nitrogen (BUN) [11, 12], and creatinine [11, 12] The dosage of acetaminophen was also investigated separately from the above risk factors. As per the guidelines, intravenous administration of 1,000 mg of acetaminophen is recommended for patients with acute abdomen, which is considered a high dose. In this study, we categorized patients into two: the high-dose group (≥ 1000 mg per infusion) and the low-dose group (< 1000 mg per infusion).

### Laboratory assessments

Laboratory data were classified according to the blood test reference values established by Tosei General Hospital. Specifically, serum bilirubin was categorized as ≥ 2.0 mg/dL, albumin as ≤ 3.5 g/dL, BUN as ≥ 20 mg/dL, and creatinine as ≥ 2.0 mg/dL. Patients were then divided into two groups based on these cutoff values.

### Statistical analyses

Univariate analysis was first conducted using Fisher's exact test to assess the relationship between post-admission delirium onset and various patient characteristics. The variables included sex, acetaminophen intravenous dose (≥ 1,000 mg/administration vs. < 1,000 mg/administration), presence of neurodegenerative diseases/dementia, history of delirium, sensory impairments (vision/hearing), and serum albumin level (≤ 3.5 g/dL vs. > 3.5 g/dL). Variables with a *p*-value of < 0.25 in the univariate analysis were selected for multivariate analysis. A multivariate logistic regression analysis was then performed with delirium onset as the dependent variable and the selected factors as independent variables to adjust for confounding factors. The impact of high-dose acetaminophen on the onset of delirium was evaluated using the multivariate logistic regression model. A *p*-value of < 0.05 was considered statistically significant. All statistical analyses were performed using IBM SPSS Statistics for Windows version 28.0 (IBM Corp., Armonk, NY, USA).

### Ethical considerations

This study was conducted in accordance with the ethical principles of the Declaration of Helsinki. Ethical approval was obtained from the Ethics Committee of Tosei General Hospital (Approval No.: 1180) and the Ethics Committee of Nagoya City University (Approval No.: 60–24-0024).

## Results

**Fig. 1:**
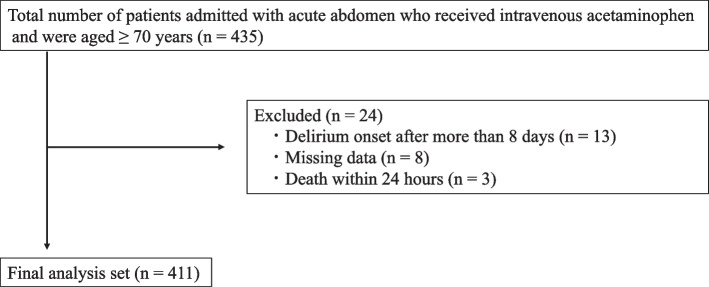
Flowchart of patient selection and exclusion criteria. Outlines the flowchart used for selecting and excluding patients from the study

**Fig. 2:**
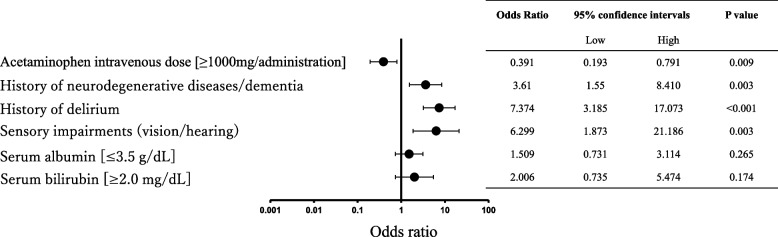
Forest Plot of Factors Associated with Delirium Onset in Patients Receiving High-Dose Intravenous Acetaminophen. The relationship between several factors and the onset of delirium in elderly patients receiving high-dose intravenous acetaminophen (≥ 1,000 mg/administration). Odds ratios (ORs) and 95% confidence intervals (CIs) are presented for each factor, including history of neurodegenerative diseases/dementia, prior delirium episodes, sensory impairments, serum albumin levels (≤ 3.5 g/dL), and serum bilirubin levels (≥ 2.0 mg/dL). ORs greater than 1 indicate an increased risk of delirium, while ORs less than 1 suggest a protective effect

The patient characteristics are summarized in Table 1. A total of 435 patients were initially included in this study. Of these, 3 patients who died within 24 h, 8 patients with missing data, and 13 patients in whom delirium onset occurred over 7 days post-admission were excluded. Consequently, 411 patients were included in the final analysis. The flowchart of patient selection is shown in Fig. 1. The patients were divided into two groups based on the presence or absence of delirium onset (Table 2). Among the 411 patients included in this study, 53 developed delirium (12.9%). In the univariate analysis, factors with a *p*-value of less than 0.25, including sex [male], acetaminophen intravenous dose (≥ 1,000 mg/administration), history of neurodegenerative diseases/dementia, history of delirium, sensory impairments (vision/hearing), serum albumin (≤ 3.5 g/dL), and serum bilirubin (≥ 2.0 mg/dL), were selected as variables for the multivariate logistic regression analysis. The multivariate analysis showed that high-dose intravenous acetaminophen (1,000 mg) significantly reduced the risk of delirium onset, with an odds ratio of 0.391 and a 95% confidence interval of 0.193–0.791 (Fig. 2).

**Table 1:** Patient background

	**n = 411**
Age (years)	79.7 ± 6.3
Sex [Male]	225 (54.7%)
Arrival by ambulance	218 (53.0%)
Living situation	
alone	69 (16.8%)
with family	319 (77.6%)
facility	23 (5.6%)
History of neurodegenerative diseases/dementia	41 (10.0%)
Sensory impairments (vision/hearing)	15 (3.6%)
History of delirium	39 (9.5%)
Use of drugs associated with delirium risk	119 (29.0%)
Use of 6 or more medications	186 (45.3%)
Serum bilirubin (mg/dL)	1.1 ± 0.7
Serum albumin (g/dL)	3.8 ± 0.6
Blood urea nitrogen (BUN) (mg/dL)	20.8 ± 11.4
Creatinine (mg/dL)	1.1 ± 1.1

**Table 2:** Association between delirium onset and patient characteristics

	Delirium Onset		*P*-value
	Yes (n = 53)	No (n = 358)	
Sex [Male]	25 (47.2%)	198 (55.3%)	0.302
Acetaminophen intravenous dose [≥ 1000 mg/administration]	31 (58.5%)	274 (76.5%)	0.007
History of neurodegenerative diseases/dementia	19 (35.8%)	23 (6.4%)	< 0.001
History of delirium	20 (37.7%)	19 (5.3%)	< 0.001
Sensory impairments (vision/hearing)	5 (9.4%)	10 (2.8%)	0.032
Use of antibiotics	34 (64.2%)	215 (60.1%)	0.652
Use of drugs associated with delirium risk	17 (32.1%)	102 (28.5%)	0.627
Serum albumin [≤ 3.5 g/dL]	18 (34.0%)	90 (25.1%)	0.183
Serum bilirubin [≥ 2.0 mg/dL]	8 (15.1%)	32 (8.9%)	0.209

Data are presented as n (%) or mean ± S.D.

Drugs associated with delirium risk: opioids, benzodiazepine receptor agonists, steroids, antiepileptic drugs, histamine H1 receptor antagonists, histamine H2 receptor antagonists, anticholinergics, and antipsychotics.

Data are presented as number of patients (%). Statistical analysis was performed using the Chi-square test for categorical variables.

## Discussion

In this study, we investigated whether high-dose intravenous acetaminophen administration in patients aged ≥ 70 years diagnosed with acute abdomen in the ED, could suppress the onset of delirium after hospitalization. The results showed that high-dose acetaminophen administration significantly reduced the risk of delirium onset after admission (Fig. 2). This finding aligns with previous basic research suggesting that acetaminophen acts on neural cells in the brain [10] and with experimental studies indicating its impact on mental state [9]. While previous studies have reported that oral administration of acetaminophen affects emotional regulation in young, healthy individuals [9], this study is the first to demonstrate the effect of high-dose acetaminophen administration in suppressing delirium onset in older patients with acute abdomen. These results suggest that high-dose acetaminophen may be effective for pain management and delirium prevention.

The incidence of delirium is known to vary, depending on age and underlying conditions [6, 13]. In our study, despite previous reports indicating a delirium incidence rate of 10–42% in hospitalized patients aged ≥ 65 years [14], high-dose acetaminophen administration was associated with a reduction in delirium onset, making this an important clinical finding. Although non-pharmacological interventions are recommended for delirium prevention [6, 15], no pharmacological therapy has been approved for the prevention of delirium. Therefore, the use of high-dose acetaminophen in pain management in the ED may contribute to the prevention of delirium onset in older patients.

Regarding acetaminophen dosage, the mean dose per kilogram of body weight was 18.2 ± 3.3 mg/kg in the group receiving 1,000 mg and 14.5 ± 2.3 mg/kg in the lower-dose group. Further research is required to categorize the dosage per kilogram more precisely to identify the most effective dose for suppressing delirium onset. In this study, the proportion of patients who developed delirium was significantly lower in the high-dose acetaminophen group. These findings suggest that high-dose acetaminophen may contribute to the suppression of delirium onset. Such an observation is consistent with results from studies such as Subramaniam et al. [16], which demonstrated acetaminophen’s effect on reducing delirium in postoperative cardiac surgery patients.

This study has some limitations. First, medical staff made the determination of delirium onset and diagnoses were not confirmed by psychiatrists, which may have limited diagnostic accuracy. Second, medical staff may have implemented additional precautions to prevent delirium in high-risk patients, potentially influencing the results.

## Conclusion

Administering high-dose intravenous acetaminophen to elderly patients with acute abdomen in the ED was associated with a reduced incidence of post-admission delirium. By highlighting the potential effectiveness of acetaminophen in preventing delirium onset, this study enhances the safety of both patients and medical staff.

## Data Availability

No datasets were generated or analysed during the current study.

## Abbreviations

BUN: Blood urea nitrogen
ED: Emergency Department
